# An integrated set-up for ex vivo characterisation of biaxial murine artery biomechanics under pulsatile conditions

**DOI:** 10.1038/s41598-021-81151-5

**Published:** 2021-01-29

**Authors:** Myrthe M. van der Bruggen, Koen D. Reesink, Paul J. M. Spronck, Nicole Bitsch, Jeroen Hameleers, Remco T. A. Megens, Casper G. Schalkwijk, Tammo Delhaas, Bart Spronck

**Affiliations:** 1 Department of Biomedical Engineering, CARIM School for Cardiovascular Diseases, Maastricht University, Universiteitssingel 50, Room 3.359, 6229ER Maastricht, The Netherlands; 2 Innovatest Europe BV, Maastricht, The Netherlands; 3 Muroidean Facility, CARIM School for Cardiovascular Diseases, Maastricht University, Maastricht, The Netherlands; 4 Institute for Cardiovascular Prevention, Ludwig-Maximilians-Universität, Munich, Germany; 5 Department of Internal Medicine, CARIM School for Cardiovascular Diseases, Maastricht University Medical Centre+, Maastricht, The Netherlands; 6 Department of Biomedical Engineering, School of Engineering & Applied Science, Yale University, New Haven, CT USA

**Keywords:** Biomedical engineering, Cardiovascular biology

## Abstract

Ex vivo characterisation of arterial biomechanics enables detailed discrimination of the various cellular and extracellular contributions to arterial stiffness. However, ex vivo biomechanical studies are commonly performed under quasi-static conditions, whereas dynamic biomechanical behaviour (as relevant in vivo) may differ substantially. Hence, we aim to (1) develop an integrated set-up for quasi-static and dynamic biaxial biomechanical testing, (2) quantify set-up reproducibility, and (3) illustrate the differences in measured arterial stiffness between quasi-static and dynamic conditions. Twenty-two mouse carotid arteries were mounted between glass micropipettes and kept fully vasodilated. While recording pressure, axial force (*F*), and inner diameter, arteries were exposed to (1) quasi-static pressure inflation from 0 to 200 mmHg; (2) 300 bpm dynamic pressure inflation (peaking at 80/120/160 mmHg); and (3) axial stretch (λ_*z*_) variation at constant pressures of 10/60/100/140/200 mmHg. Measurements were performed in duplicate. Single-point pulse wave velocities (PWV; Bramwell-Hill) and axial stiffness coefficients (*c*_ax_ = d*F*/dλ_*z*_) were calculated at the in vivo value of λ_*z*_. Within-subject coefficients of variation were ~ 20%. Dynamic PWVs were consistently higher than quasi-static PWVs (*p* < 0.001); *c*_ax_ increased with increasing pressure. We demonstrated the feasibility of ex vivo biomechanical characterisation of biaxially-loaded murine carotid arteries under pulsatile conditions, and quantified reproducibility allowing for well-powered future study design.

## Introduction

Large artery stiffness is a strong and independent predictor for cardiovascular events as well as all-cause mortality in the general population^1–5^. Therefore, treatment of arterial stiffness is considered to be a good target in prevention of cardiovascular disease^6–9^. Clinical arterial stiffness measurements, however, do not yield direct insight in the underlying biomechanics of the arterial wall. To enable more detailed assessment of biomechanical properties, ex vivo experimental studies are useful. Ex vivo characterisation allows more tightly controlled hemodynamic and mechanical conditions, enabling discrimination of e.g. cellular and extracellular processes in vascular remodelling^10^. Importantly, in contrast to in vivo measurements, ex vivo studies allow biomechanical characterisation beyond the physiological pressure range. Moreover, biaxial data on pressure, diameter, length, and axial force of the artery can be recorded simultaneously during ex vivo experiments. Integration of the data using a computational model of arterial wall mechanics is useful to obtain quantitative insight into the constitutive properties of arteries^11,12^.

Whereas most ex vivo experiments on arteries are performed under quasi-static conditions (i.e. slowly increasing pressure), cyclic stretch at a physiological rate emerged as a major determinant of vascular function and mechanical homeostasis^7,13–16^. Previous studies imply that arterial biomechanical behaviour may substantially differ under dynamic and static conditions^16–20^. A number of studies have performed ex vivo dynamic assessment of large artery stiffness and distensibility, in e.g. rat^21^ and pig^22^. To our knowledge, the first study subjecting biaxially loaded murine carotid arteries to pulsatile pressure was by Gleason et al.^10^. However, pressure waves in their set-up were sinusoidal and the sites of pressure measurement were distant from the mounting pipettes. Furthermore, because of the pipette impedances and nonzero flow through them, a pressure measured through a narrow pipette will exhibit a (frequency-dependent) phase difference with measured diameter, which potentially hampers correct identification of viscous and elastic contributions to artery wall stress–strain behaviour^23^.

A potential solution to this problem is to employ a wire myography-based technique, as performed by Leloup et al.^7^. Although using such technique, circumferential force and displacement can be directly measured, axial stretch cannot be manipulated, prohibiting the study of the axial biomechanical behaviour. Furthermore, because of the lack of axial constraint, also during circumferential measurements, vessels will not be held at an in vivo relevant axial stretch (${\uplambda }_{z}$; in the order of ~ 1.7 for carotid arteries). Instead, in wire myography, the axial stretch during measurement will typically be *smaller* than unity (${\uplambda }_{z} < 1$, i.e. the vessels are axially compressed instead of stretched). This non-physiological axial stretching state has important functional consequences: e.g. it influences sensitivity to vasoactive substances^24^, but also directly influences measured circumferential properties due to axial-circumferential coupling^25^.

In the present study, we describe and characterise a set-up for integrated biomechanical characterisation of biaxially loaded passive (i.e. without vascular smooth muscle cell (VSMC) contribution) murine carotid arteries under pulsatile as well as quasi-static conditions, closely mimicking in vivo conditions. Specifically, we aim to (1) present the details of our set-up, (2) quantify set-up reproducibility, and (3) illustrate the difference in measured arterial stiffness between testing under quasi-static and dynamic conditions.

**Figure 1:**
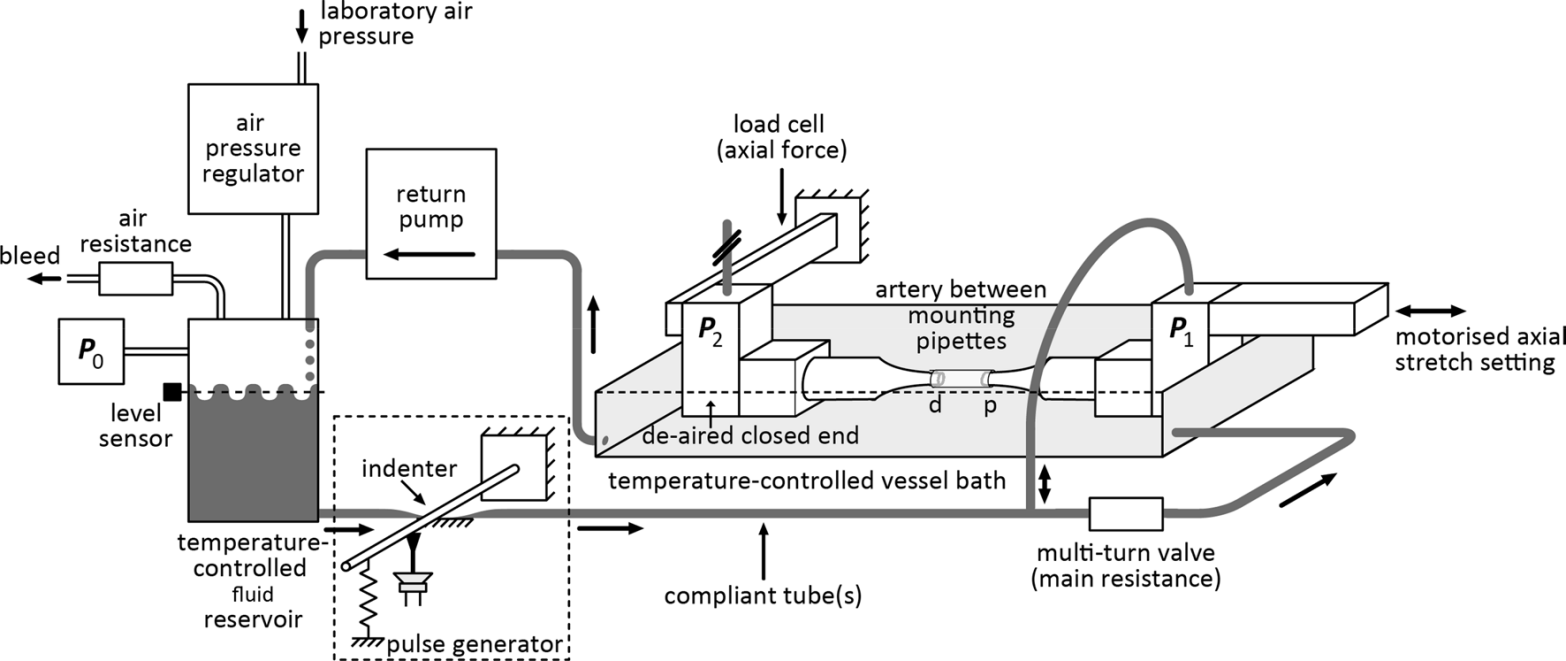
Set-up specialised for biaxial biomechanical assessment of murine arteries under pulsatile conditions. p and d, proximal and distal pipettes for mounting the artery. *P*_0_, *P*_1_, and *P*_2_ are sensors registering reservoir and proximal and distal pipette pressures, respectively. Note that sensor *P*_2_ is at a de-aired closed end. For detailed description, see main text.

The set-up presented herein has a pipette and pressure recording configuration that avoids the aforementioned frequency-dependent phase errors. By closing and de-airing the outflow end of the system, there was no flow across the distal pipette (Fig. 1). The distal pipette acts as an extension of the pressure sensor, ultimately eliminating the problem of pipette impedance*.* Vessels with a typical loaded outer diameter of 0.7 mm can be tested, making our set-up highly suitable for biomechanical characterisation of (large) arteries of the mouse, an animal model abundant in the field. Inflation experiments under quasi-static as well as dynamic (i.e. close to in vivo) conditions at pressures ranging from 0 to 200 mmHg and a pulse frequency of 5 Hz (300 bpm) are performed with the vessel at or around its in vivo value of axial stretch, yielding relevant loading conditions. In addition, axial extension measurements at fixed pressure levels allow for quantification of axial mechanical behaviour.

## Results

### Choice of pressure measurement location to represent intravascular pressure

**Figure 2:**
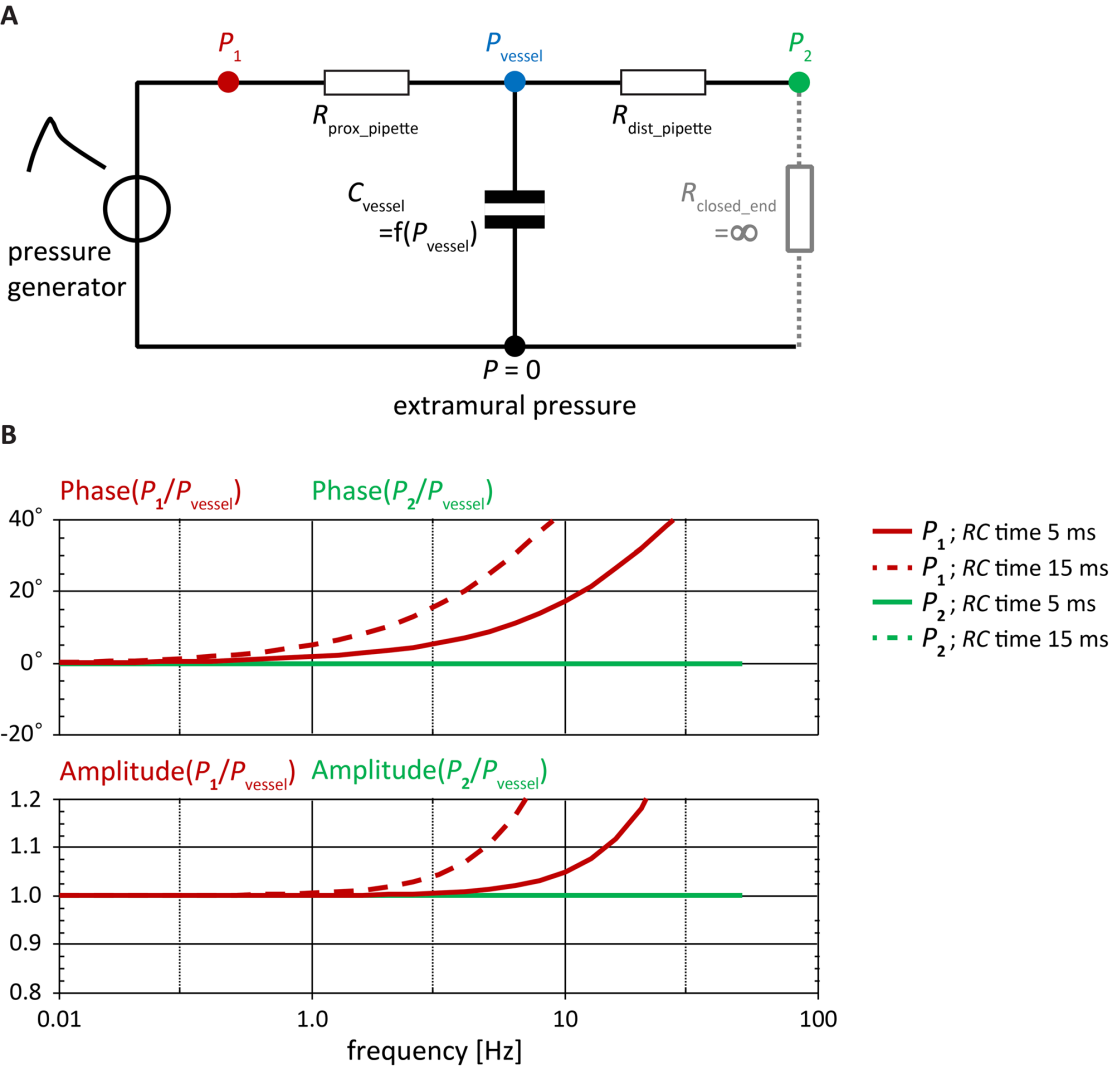
(**A**) Schematic model of the pipette configuration and pressure recording positions. *P*_1_ indicates the proximal pressure sensor; *P*_2_ indicates the distal pressure sensor; and *P*_vessel_ indicates the true pressure inside the vessel under test. *R*_closed_end_ models the stopcock distal to sensor *P*_2_ which during measurements is closed to prevent any flow through the distal pipette (modelled by *R*_dist_pipette_). We used the “*C*_vessel_ = f (*P*_vessel_)” notation to indicate that *C*_vessel_ is strongly dependent on transmural pressure (due to nonlinear arterial elasticity). (**B**) Phase (top) and amplitude (bottom) bode diagrams, showing amplitude relationships of *P*_2_ and *P*_1_ to *P*_vessel_, as a function of frequency. A phase of zero degrees indicates no phase error; an amplitude of 1.0 indicates no amplitude error. Continuous lines indicate the situation for an *RC*-time (defined as *R*_prox_pipette_∙*C*_vessel_) of about 5 ms and the dashed lines for a tripled *RC*-time (i.e. 15 ms, with *C*_vessel_ assumed 3 times larger and *R*_prox_pipette_ kept constant). *P*_1_ shows considerable, frequency-dependent errors, whereas *P*_2_ shows flat curves with no errors in the relevant frequency range (i.e. 5 Hz cycle times 10 harmonics to represent the waveform requires at least 50 Hz). Taken together, pressure as recorded at *P*_2_ is representative of *P*_vessel_. In contrast, *P*_1_ shows a large, frequency and compliance-dependent difference (error) from *P*_vessel_.

**Figure 3:**
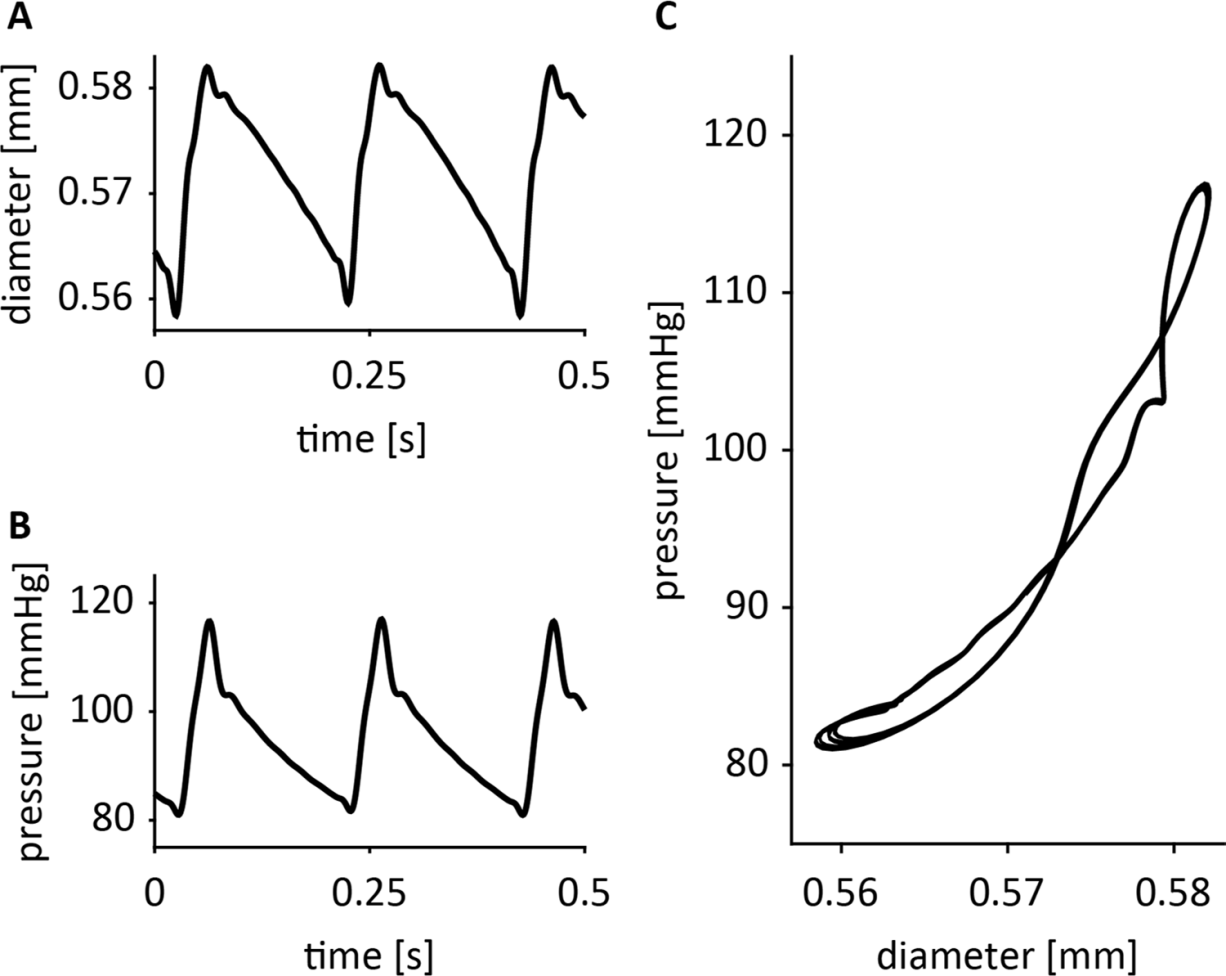
Representative examples of inner diameter (**A**) and pressure (**B**) tracings, and the resulting pressure-diameter plot (**C**), exposing considerable curvilinearity but rather limited loop area.

The pipette configuration we used (Fig. 1) is essential for obtaining dynamic pressure-diameter curves with negligible phase- and amplitude errors. We chose to use one pipette (proximal (p) in Fig. 1) to inflate/deflate the artery, and the other pipette (distal (d)) to obtain a representative intravascular pressure measurement at sensor *P*_2_. Lumped-parameter modelling simulations indeed showed no phase error between intravascular pressure (*P*_vessel_) and *P*_2_ for the reference situation (at an *RC* time of 5 ms, typical for our set-up and a control carotid artery), whereas *P*_1_ showed a frequency-dependent phase error of 2 degrees at 1 Hz up to 18 degrees at 10 Hz (continuous lines; Fig. 2). Correspondingly, the amplitude error remains zero for *P*_2_, whereas for *P*_1_ e.g. a 5% overestimation is attained at 10 Hz. Importantly, with a threefold increase in vessel compliance (dashed lines; *RC* time = 15 ms) the phase and amplitude errors for *P*_1_ increase, whereas those for *P*_2_ remain unaffected (Fig. 2). It should be noted that because our set-up generates physiological pressure waveforms (cf. Fig. 3) the relevant bandwidth is about 50 Hz (i.e. 10 harmonics at a base frequency of 5 Hz). Hence, if *P*_1_ would be used to represent *P*_vessel_, a substantial overestimation and phase error would occur at frequencies within this relevant bandwidth. When using *P*_2_ to represent *P*_vessel_, these problems are negligible.

### Set-up reproducibility

Within-subject coefficients of variation (CVs) for quasi-static and dynamic pulse wave velocity (PWV) and axial stiffness coefficient were approximately 20%. Detailed reproducibility statistics are presented in Table 1.

**Table 1:** Repeatability statistics.

	*n*_sample_	Mean	Standard deviation (SD) and coefficient of variation (CV)				
				Within-subject	Between-subject	Composite (*n*_rep_ = 1)	Composite (*n*_rep_ = 2)
**Dynamic inflation pulse wave velocity (PWV)**							
80 mmHg	19	2.43 (2.38–2.49) m/s	SD	0.43 (0.30–0.50) m/s	0.17 (0.09–0.20) m/s	0.46 (0.35–0.53) m/s	0.35 (0.28–0.38) m/s
			CV	17.5 (12.5–20.4) %	7.1 (3.7–8.3) %	18.9 (14.4–21.2) %	14.3 (11.6–15.5) %
120 mmHg	19	6.00 (5.83–6.16) m/s	SD	1.25 (1.11–1.37) m/s	0.65 (0.07–0.83) m/s	1.41 (1.28–1.48) m/s	1.10 (0.93–1.18) m/s
			CV	20.9 (18.4–22.9) %	10.8 (1.1–13.9) %	23.5 (21.3–24.8) %	18.3 (15.4–19.7) %
160 mmHg	18	13.49 (13.12–13.86) m/s	SD	2.28 (2.10–2.44) m/s	1.75 (0.92–2.06) m/s	2.87 (2.45–3.06) m/s	2.38 (1.83–2.59) m/s
			CV	16.9 (15.4–18.4) %	13.0 (6.2–15.4) %	21.3 (17.9–23.4) %	17.6 (13.2–20.0) %
**Quasi-static inflation pulse wave velocity (PWV)**							
80 mmHg	18	2.15 (2.10–2.20) m/s	SD	0.37 (0.23–0.45) m/s	0.14 (0.05–0.18) m/s	0.40 (0.24–0.49) m/s	0.30 (0.18–0.36) m/s
			CV	17.3 (11.0–20.5) %	6.7 (2.2–8.2) %	18.6 (11.6–21.8) %	14.0 (8.6–15.9) %
120 mmHg	18	4.93 (4.81–5.05) m/s	SD	1.19 (0.90–1.37) m/s	N/A* (N/A-0.37) m/s	1.15 (1.00–1.23) m/s	0.78 (0.69–0.81) m/s
			CV	24.2 (18.4–27.5) %	N/A (N/A-7.6) %	23.2 (20.3–24.8) %	15.7 (14.1–16.4) %
160 mmHg	17	10.77 (10.52–11.02) m/s	SD	1.91 (1.73–2.05) m/s	0.86 (0.19–1.08) m/s	2.09 (1.93–2.17) m/s	1.60 (1.40–1.68) m/s
			CV	17.7 (15.9–19.3) %	8.0 (1.8–10.0) %	19.4 (17.8–20.3) %	14.9 (13.0–15.6) %
**Quasi-static axial stiffness coefficient (c**_**ax**_**)**							
10 mmHg	19	1.27 (1.24–1.30) g	SD	0.25 (0.22–0.27) g	0.09 (N/A-0.13) g	0.26 (0.24–0.28) g	0.20 (0.17–0.21) g
			CV	19.6 (17.0–21.6) %	6.8 (N/A-10.1) %	20.8 (19.0–21.7) %	15.4 (13.4–16.3) %
60 mmHg	19	1.30 (1.27–1.33) g	SD	0.26 (0.24–0.29) g	0.07 (N/A-0.11) g	0.27 (0.23–0.30) g	0.20 (0.15–0.22) g
			CV	20.3 (18.3–21.9) %	5.1 (N/A-8.4) %	20.9 (18.1–22.5) %	15.2 (11.7–16.6) %
100 mmHg	19	2.65 (2.58–2.71) g	SD	0.38 (0.32–0.43) g	0.35 (0.28–0.38) g	0.52 (0.46–0.55) g	0.44 (0.39–0.47) g
			CV	14.4 (12.1–16.0) %	13.4 (10.8–14.5) %	19.6 (17.5–20.7) %	16.8 (14.8–17.6) %
140 mmHg	19	5.27 (5.09–5.45) g	SD	0.46 (0.40–0.52) g	1.13 (0.96–1.22) g	1.23 (1.07–1.30) g	1.18 (1.01–1.26) g
			CV	8.8 (7.6–9.7) %	21.5 (18.3–22.9) %	23.3 (20.5–24.4) %	22.4 (19.4–23.7) %
200 mmHg	19	10.48 (10.13–10.83) g	SD	0.84 (0.77–0.90) g	2.26 (1.93–2.41) g	2.41 (2.11–2.55) g	2.33 (2.02–2.48) g
			CV	8.1 (7.4–8.6) %	21.5 (18.6–22.8) %	23.0 (20.3–24.1) %	22.3 (19.4–23.5) %

*n*_sample_: number of included samples. Only samples which were measured in duplicate are included in this analysis (hence 2*n*_sample_: number of included measurements). Values between parentheses indicate the 25–75% confidence interval, obtained using (nonparametric) percentile bootstrapping. Within-subject and between-subject SDs based on analysis described by Rodbard^67^. *N/A* not applicable; imaginary number arising from a negative estimated between-subject variance caused by sampling error. Composite SDs (*s*) represent the expected ‘between-sample variation’ for an unknown sample of *n*_rep_ replicates, and were calculated using $s = \sqrt {s_{{\text{b}}}^{2} + s_{{\text{w}}}^{2} /n_{{{\text{rep}}}} }$, with $s_{{\text{b}}}$ and $s_{{\text{w}}}$ the between- and within-subject SD, respectively. This equation can also be used to estimate expected SDs for any *n*_rep_, which may be useful for power calculations for future studies.

*For quasi-static PWV at 120 mmHg, $s_{{\text{b}}}^{2} = - \;0.11{\text{ m}}^{2} /{\text{s}}^{2}$.

### Quasi-static inflation

**Figure 4:**
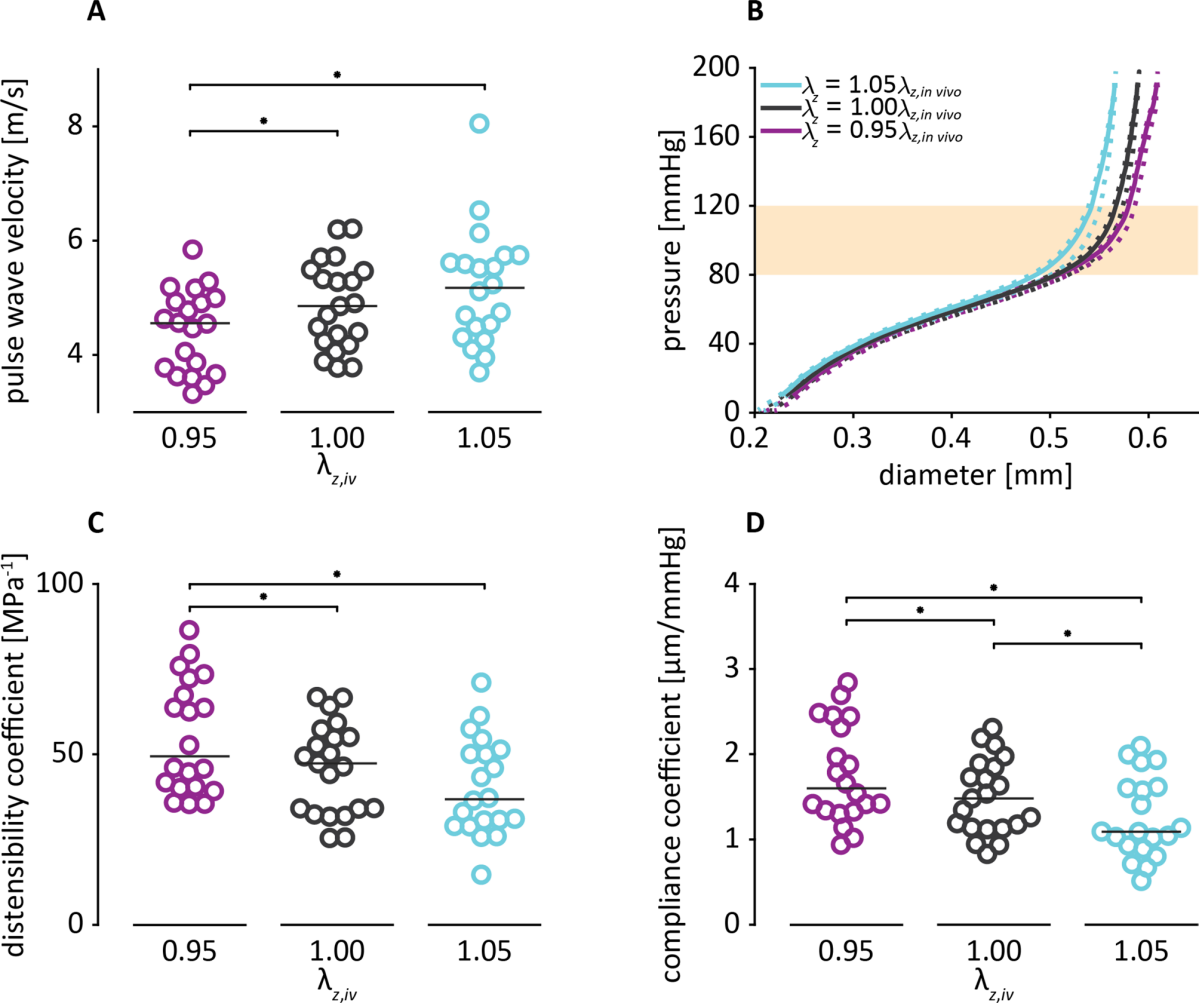
Quasi-static arterial stiffness increased with increasing stretch. (**A**) pulse wave velocity (PWV) determined from quasi-static diameter relationships for different stretch ratios. PWV was determined at 120/80 mmHg (orange area in (**B**)). Lines indicate median PWV. (**B**) Representative example of quasi-static pressure-diameter relationships from one vessel at different stretch ratios. Dashed lines reflect the pressure-diameter relation during inflation (ascending line) and deflation (descending line), the straight line reflects the average. (**C**) Distensibility coefficient (DC) determined from quasi-static pressure-diameter curve at reference pressure 120/80 mmHg. (**D**) Compliance coefficient (CC) determined from quasi-static pressure-diameter curve at reference pressure 120/80 mmHg. *p < 0.05, *n* = 20.

Axial stretch significantly influenced quasi-static PWV measured at pressure range 120/80 mmHg (χ^2^(2) = 28.3, *p* < 0.001). Dunn-Bonferroni post hoc testing revealed PWV at ${\uplambda }_{z} = 0.95{\uplambda }_{z,iv}$ (4.6 [3.7–5.0] m/s) to be significantly lower than at ${\uplambda }_{z} = {\uplambda }_{z,iv}$ (4.9 [4.2–5.5] m/s, *p* < 0.001) and ${\uplambda }_{z} = 1.05{\uplambda }_{z,iv}$ (5.2 [4.4–5.7] m/s, *p* < 0.001; Fig. 4A/B), with ${\uplambda }_{z,iv}$ indicating the estimated axial stretch that the artery was at in vivo (see “Methods” section).

Similar results were found for quasi-static distensibility coefficient (DC) measured at the pressure range of 120/80 mmHg (*χ*^2^(2) = 25.9, p < 0.001). Post hoc testing revealed a significant difference between $\lambda_{z} = 0.95\lambda_{z,iv}$ and $\lambda_{z} = \lambda_{z,iv}$ (49.4 [40.3–69.8] vs. 47.3 [33.6–55.6], MPa^−1^, p = 0.001). This significant difference was also found between $\lambda_{z} = 0.95\lambda_{z,iv}$ and $\lambda_{z} = 1.05\lambda_{z,iv}$ (36.8 [29.8–50.7], MPa^−1^, p < 0.001; Fig. 4C).

Finally, these results were confirmed in quasi-static compliance coefficient (CC) outcomes as well (*χ*^2^(2) = 34.3 *p* < 0.001). Again, CC at $\lambda_{z} = 0.95\lambda_{z,iv}$ (1.54 [1.32–2.35] µm/mmHg) to be significantly higher compared to $\lambda_{z} = \lambda_{z,iv}$ (1.48 [1.14–1.86] µm/mmHg, *p* = 0.005) and $\lambda_{z} = 1.05\lambda_{z,iv}$ (1.09 [0.91–1.61] µm/mmHg, *p* < 0.001; Fig. 4). $\lambda_{z} = \lambda_{z,iv}$ and $\lambda_{z} = 1.05\lambda_{z,iv}$ also significantly differed in this analysis (*p* = 0.022; Fig. 4D)*.*

### Dynamic (pulsatile) inflation

**Figure 5:**
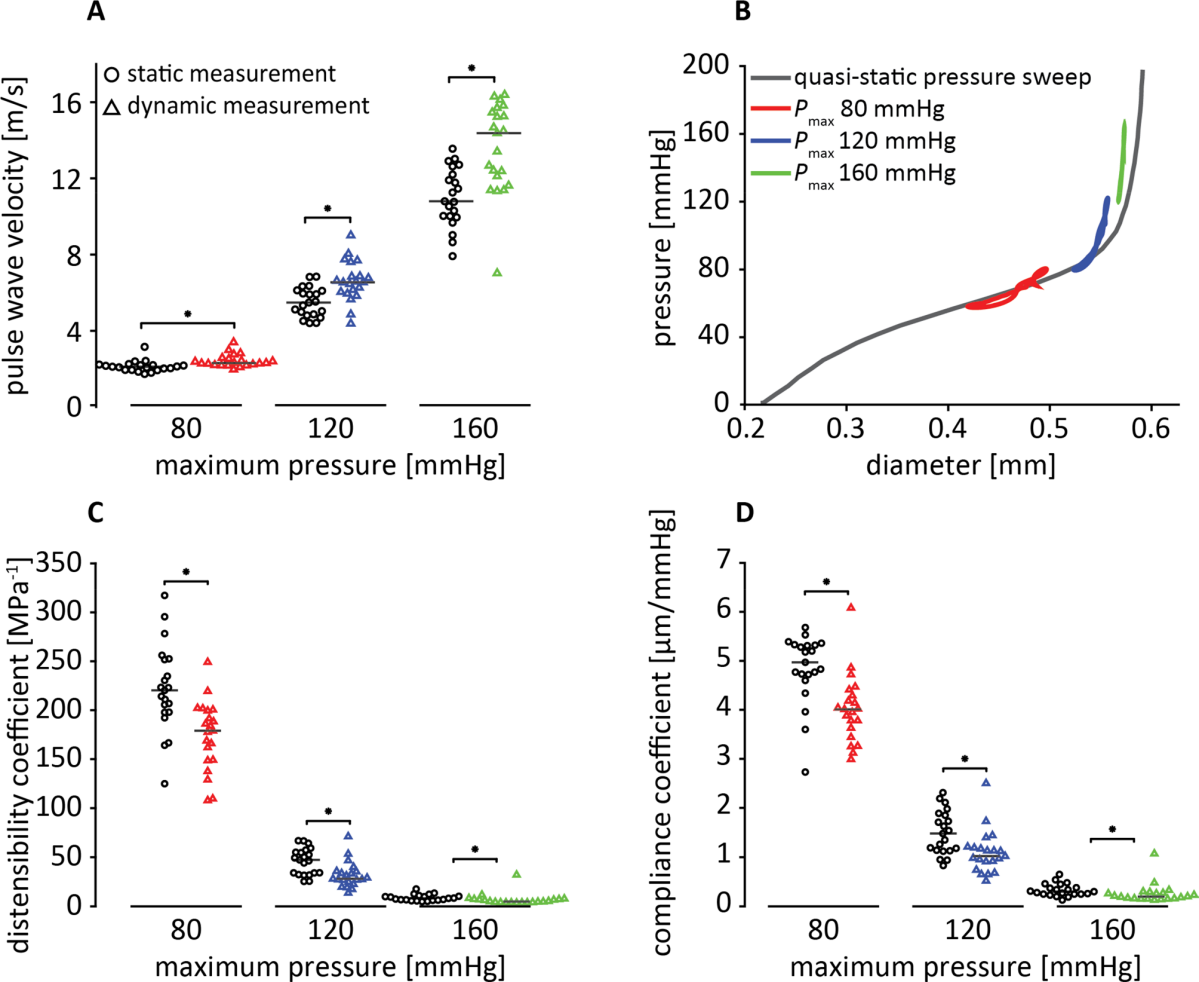
Static experiments underestimate stiffness determined under pulsatile (in vivo) conditions. (**A**) PWV determined from dynamic pressure-diameter curves compared to the corresponding quasi-static pressure-diameter curve. (**B**) Representative set of quasi-static (grey) and dynamic pressure-diameter curves (the latter obtained for maximum pressures *P*_max_ of 80, 120, 160 mmHg). (**C**) Distensibility coefficient (DC) determined from dynamic pressure-diameter curves compared to the corresponding quasi-static pressure-diameter curve. (**D**) Compliance coefficient (CC) determined from dynamic pressure-diameter curves compared to the corresponding quasi-static pressure-diameter curve. *p < 0.05, *n* = 21.

Dynamic PWV measured at 5 Hz (300 bpm) was consistently higher than quasi-static PWV for 80 mmHg (2.3 [2.2–2.5] vs. 2.1 [1.9–2.2] m/s, *p* < 0.001), 120 mmHg (5.9 [5.4–6.4] vs. 4.9 [4.2–5.5] m/s, *p* < 0.001), and 160 mmHg (14.4 [11.6–15.5] vs. 10.8 [9.9–12.3] m/s, *p* = 0.001; Fig. 5A,B). As expected, PWV showed an increase with increasing transmural pressure for both the dynamic and quasi-static measurements (χ^2^(2) = 40.1, *p* < 0.001; χ^2^(2) = 42.0, *p* < 0.001).

In line with PWV results, DC was higher in quasi-static measurements compared to dynamic measurements for 80 mmHg (220.2 [197.9–251.9] vs. 178.9 [149.0–199.6] MPa^−1^, *p* < 0.001), 120 mmHg (47.3 [33.6–55.6] vs. 28.1 [24.3–35.5] MPa^−1^, *p* < 0.001), and 160 mmHg (8.3 [6.4–10.5] vs. 5.0 [3.9–7.3] MPa^−1^, *p* = 0.001; (Fig. 5C)). DC showed a decrease with increasing transmural pressure for both the dynamic and quasi-static measurements (*χ*^2^(2) = 40.1, *p* < 0.001; *χ*^2^(2) = 42.0, *p* < 0.001).

As expected, CC was also higher in quasi-static compared to dynamic measurements for 80 mmHg (4.97 [4.69–5.32] vs. 4.01 [3.58–4.32], µm/mmHg, *p* = 0.002), 120 mmHg (1.48 [1.14–1.86] vs. 1.02 [0.87–1.20], µm/mmHg, *p* < 0.001), and 160 mmHg (0.30 [0.25–0.40] vs. 0.20 [0.16–0.28], µm/mmHg, *p* = 0.004 (Fig. 5D)). CC decreased with increasing transmural pressure for both the dynamic and quasi-static measurements (*χ*^2^(2) = 40.1, *p* < 0.001; *χ*^2^(2) = 42.0, *p* < 0.001).

### Quasi-static axial extension

**Figure 6:**
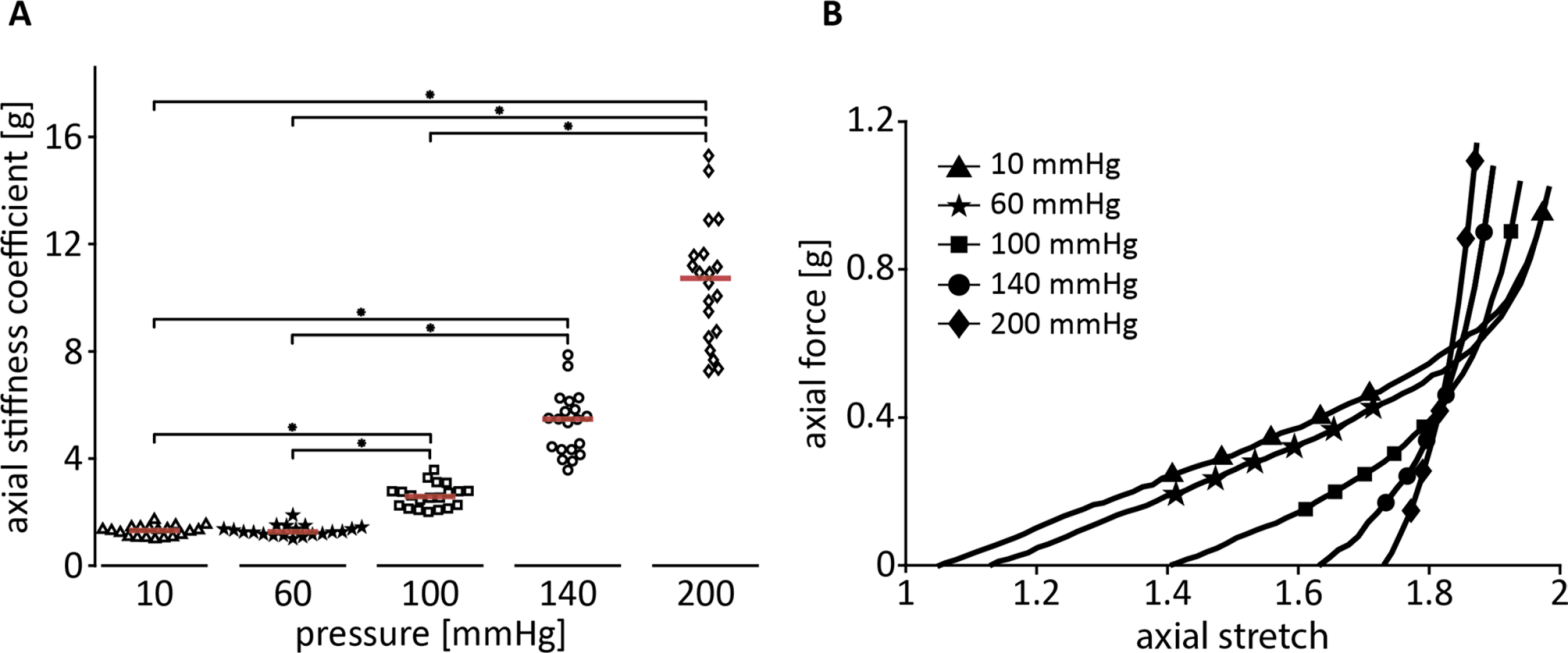
Axial stiffness coefficient increases with increasing pressure. (**A**) Axial stiffness coefficient was determined as the local derivative at ${\uplambda }_{z} = {\uplambda }_{z,iv}$ (mean crossing points of force-stretch curves (**B**)). Asterisks indicate statistically significant differences between groups. *p < 0.05, *n* = 20. (**B**) Representative quasi-static force-stretch relation under different static pressures.

The axial stiffness coefficient increased significantly with increasing pressure (χ^2^(4) = 76.2, *p* < 0.001). Dunn-Bonferroni post hoc tests revealed the axial stiffness coefficient increased with increasing pressure (1.3 [1.1–1.4], 1.3 [1.2–1.4], 2.6 [2.2–2.8], 5.5 [4.3–6.1], and 10.7 [8.6–11.6] g, respectively) between all groups (*p* < 0.05) but not between 10 and 60, 100 and 140, and 140 and 200 mmHg (*p* = 1.00, *p* = 0.46 and *p* = 0.46 respectively; Fig. 6).

### Influence of overnight storage

The maximum time between isolation and mechanical testing of the common carotid artery (CCA) was 24 h in this study. We did not observe any influence of storage up to 24 h on arterial mechanics (Tables S1–S3). In particular, we did not find a difference in quasi-static PWV between fresh and non-fresh tested vessels at different axial stretches ($0.95{\uplambda }_{z,iv}$, *p* = 0.80; $1.00{\uplambda }_{z,iv}$, *p* = 0.65; $1.05{\uplambda }_{z,iv}$, *p* = 0.052), during quasi-static inflation experiments (80 mmHg, *p* = 0.65; 120 mmHg, *p* = 1.00; 160 mmHg, *p* = 0.65), or dynamic inflation experiments (80 mmHg, *p* = 0.28; 120 mmHg, *p* = 0.86; 160 mmHg, *p* = 0.47). In addition, axial stiffness coefficients did not differ between fresh and non-fresh tested vessels (10 mmHg, *p* = 0.66; 60 mmHg, *p* = 1.00; 100 mmHg, *p* = 0.18; 140 mmHg, *p* = 0.66; 200 mmHg, *p* = 0.66).

### Difference between left and right common carotid arteries

PWV between left and right carotid arteries differed at ${\uplambda }_{z} = {\uplambda }_{z,iv}$ and 120 mmHg for quasi-static inflation experiments (4.4 [4.1–4.9] vs. 5.4 [4.7–5.8] m/s, *p* = 0.04). This difference was neither found at ${\uplambda }_{z} = 0.95{\uplambda }_{z,iv}$ (*p* = 0.58), nor at $1.05{\uplambda }_{z,iv}$ (*p* = 0.39; Table S1). There was no significant difference between left and right carotid arteries during quasi-static inflation experiments at 80 mmHg (*p* = 0.65), and 160 mmHg (*p* = 0.39; Table S2), nor during dynamic inflation experiments (80 mmHg, *p* = 0.13; 120 mmHg. *p* = 0.86; 160 mmHg, *p* = 0.70; Table S2). In addition, axial stiffness coefficients did not differ between left and right carotid arteries for all tested pressures (*p* = 0.39, *p* = 0.97, *p* = 1.00, *p* = 0.97, and *p* = 0.48; Table S3).

## Discussion

In the present study, we present and evaluate a measurement set-up for biomechanical characterisation of murine arteries under pulsatile conditions and at a realistic axial stretch. Measurements showed an acceptable reproducibility, with within-subject CVs of about 20% (Table 1). We observed a significant difference in PWV between quasi-static and dynamic measurements, as a function of pressure (Fig. 5A). This finding indicates that pulsatile (in comparison to quasi-static) loading indeed affects the biomechanical behaviour of the vessel wall.

### Dynamic vs. quasi-static experiments

With our set-up, we aimed to mimic in vivo conditions, using sharply rising pressure pulses reasonably similar to an in vivo blood pressure waveform. At 5 Hz (300 bpm), upstroke duration in our system was ~ 36 ms (Fig. 3), which better mimics the in vivo rate of circumferential deformation than, e.g., sinusoidal waveforms, where the upstroke duration would be 100 ms at 5 Hz.

It is important to note our specific pipette and pressure recording configuration (Figs. 1, 2)^18,21,26–29^. This configuration, in conjunction with the zero-phase error processing of pressure and diameter signals, is essential for interpreting observed differences between quasi-static and dynamic behaviour in terms of (viscous) wall behaviour. If the properties of the tested vessel itself would determine the phase and frequency response, the respective contributions of system and vessel could not be disentangled. This would have been the case if we had used *P*_1_ instead of *P*_2_ to represent intravascular pressure. Taken together, the design and implementation of our set-up are optimally suited to record dynamic pressure-diameter curves over the large pressure range considered using physiological pulse waveforms (see “Experimental procedures” in “Methods” section).

Our measured dynamic pressure-diameter (*P*-*d*) curves did not exhibit significant hysteresis (no substantial net loop area (Fig. 5B)). Dynamic *P*-*d* curves at lower mean pressures tended to show more loop-like behaviour, be it in ‘figure-8’ fashion, with the net loop area remaining small. The negligible loop area we observed is corroborated by *P*-*d* curves as measured in vivo in catheterised patients^30^. Although our current data do not indicate visco-elastic behaviour exposed by hysteresis in the dynamic *P-d* curves, the clear difference in overall steepness of the dynamic curves when compared to the quasi-static *P-d* curves does signify a strain rate effect, indicative of a viscous component. Others have found comparable differences between dynamic and static elastic behaviour in other experimental animals^19,20,27^.

**Figure 7:**
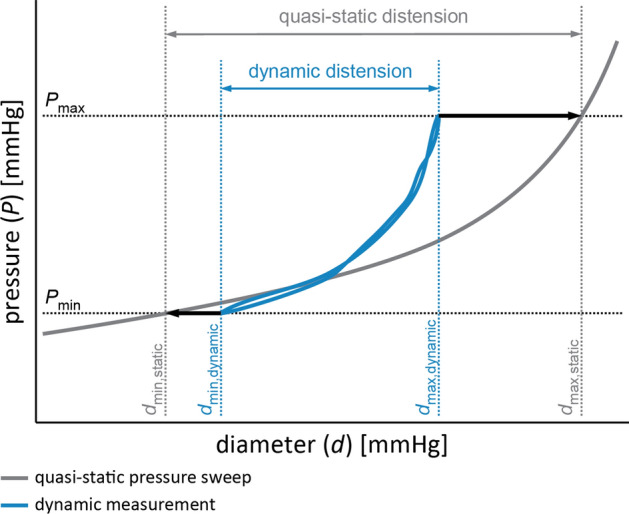
Schematic overview showing how diameters and pressures are obtained for pulse wave velocity calculation for both quasi-static and dynamic experiments. Maximum and minimum pressure (*P*_max_ and *P*_min_, respectively) are taken from the dynamic measurement. At these given pressures, the corresponding minimum and maximum diameters (*d*_min_ and *d*_max_) are obtained for the dynamic (blue line) and quasi-static (grey line) diameter recordings.

The deformation response of visco-elastic materials depends both on the amount of force applied (elastic response) and on its rate of change (viscous response)^27^. This rate of change differs between the dynamic and static inflation experiments. This translates into a smaller distension under dynamic than under static conditions, leading to a higher PWV (Eq. (1); Figs. 5A, 7). We conclude that the difference in static and dynamic PWVs is a result, at least partly, of viscous behaviour. The difference in PWV between dynamic and static experiments increases with increasing pressure (Fig. 5A). This could be due to intrinsic phenomena which are not explained by viscous behaviour only. For example, there might be residual vascular smooth muscle cell (VSMC) reactivity, which apparently is more triggered at higher pressure ranges^31,32^. Although we suppressed active contribution of VSMCs to viscous behaviour by adding sodium nitroprusside (SNP) to the buffer, this might have not been sufficient. Inactivation of VSMC by using stronger substances such as ethylenediaminetetraacetic acid (EDTA) could be performed in the future to check the effect of our current choice for SNP^33,34^.

In this study, we calculated and reported PWV based on the Bramwell-Hill equation, DC, and CC as measures of structural vascular stiffness. The use of other, large-strain stiffness metrics could potentially lead to different results and conclusions^25^. In the future, we aim to use our set-up for a full, *constitutive* characterization of arterial tissue^11^, also yielding estimates of material stiffness. Such approach necessitates calculation of stress (force per area) and hence requires a measurement of wall thickness. Wall thickness measurements can easily be obtained by cutting a ring from the artery after the experiment and measuring its thickness or cross-sectional area under a low-magnification microscope^11^. Using such thickness, outer diameter can be easily calculated from inner diameter at any stretch state (using conservation of volume). Because of the availability of pulsatile data, we will be able to extend the current standard approach (modelling the artery hyper elastically using a combination of an isotropic and fibrous component) with descriptors for visco-elasticity (e.g. quasi-linear viscoelasticity). Such studies would allow for quantification of differences in arterial viscoelasticity *on a constitutive level* in diseases such as diabetes, hypertension, or chronic kidney disease.

### Reproducibility

Our measurements showed an acceptable reproducibility, with within-subject CVs around 20% (Table 1). Sample size calculations for future studies aiming to show a treatment effect of 2 m/s result in a required *n* = 9 animals per group for dynamic PWV measurements at 120 mmHg (standard deviation (SD) = 1.41 m/s). In case measurements are performed in duplicate (as e.g. in the present study), this reduces to *n* = 6 (SD = 1.10 m/s).

In this study, the maximum time between isolation and mechanical testing of the CCA was 24 h. Although we did not perform active contraction/dilatation experiments, Gleason et al. reported that a murine carotid artery’s response to vasodilators and vasoconstrictors can be maintained for up to four days after isolation when cultured appropriately^10^.

### In vivo axial stretch determination

As the axial stretch of an artery influences the distension measured under both quasi-static and dynamic conditions, it is important to determine the in vivo axial stretch as accurately as possible^27,35^. In the present study, this in vivo axial stretch was estimated as the decoupling stretch between axial force and pressure^36,37^. In the current experimental protocol, at the in vivo axial stretch determination step, the vessel is rapidly (in about 1 s) inflated from 0 to 140 mmHg and the difference in axial force between those pressures is used to identify (deviation from) the “experimental” in vivo axial stretch ($\lambda_{z,iv}$). During data analysis (retrospectively; in silico), the in vivo axial stretch is recalculated as the mean of crossing points of the quasi-static axial force-stretch curves at 60, 100, 140, and 200 mmHg. The latter metric *(*$\lambda_{z,iv,\text{calc}}$^11, 38^) is estimated using much more data and, hence, more robust (e.g., against noise). $\lambda_{z,iv}$ and $\lambda_{z,iv,\text{calc}}$ differ slightly from each other (Fig. S1). In a future protocol, gradual inflation/deflation while recording axial force could potentially enable more precise determination of the in vivo axial stretch already at the experimental stage.

In the present study, we did not assess how $\lambda_{z,iv}$*/*$\lambda_{z,iv,\text{calc}}$ compare to the “actual” in vivo axial stretch as would be obtained from measuring the amount of retraction in situ at the time of excision ($\lambda_{z,is}$)*.* Previous studies observed that for the carotid artery, $\lambda_{z,iv}$ and $\lambda_{z,is}$ agree well^37,38^. However, this is not the case for all arteries, with e.g. Schulze-Bauer et al. observing $\lambda_{z,is}$ to be substantially smaller than $\lambda_{z,iv}$^39^ in the human iliac artery, and Jadidi et al. observing a similar distance in femoropopliteal arteries of young humans^40^. Taken together, when using our set-up to test arteries other than the carotid, care should be taken not to directly assume $\lambda_{z,iv}$*/*$\lambda_{z,iv,\text{calc}}$ to be representative of $\lambda_{z,is}$*,* to avoid the risk of tissue damage due to supraphysiological stretching and to avoid misinterpreting the obtained results as representative of the in vivo situation.

### Limitations

Under typical awake conditions, murine heart rate is ~ 600 bpm. In the current study, we performed our experiments at a pulse frequency of 5 Hz (300 bpm), which corresponds approximately to a murine heart rate under general anaesthesia^41^. As the measured biomechanical behaviour of the artery may vary with pulse frequency^42^, we aim to perform future experiments also at other pulse frequencies, e.g. 2.5 and 10 Hz.

The translation of animal experimental research to the human situation remains challenging. Murine and human arteries contain the same structures, but their pulsatile loading rates differ (500–700 bpm in mice compared to 60–100 bpm in humans). Heart rate dependency of PWV has been studied in pacing experiments in both human and rat arteries^43–45^. Relative changes in PWV that were obtained at different heart rates were very similar between the two studies, despite the different species. Tan et al. report an allometric relationship where heart rate is inversely related to body mass^44^. Such relationship could be used to translate findings for a large range of species, including murine experiments, to the human situation.

To minimise the number of animals used in research, we used surplus mice from our animal facility. This has the disadvantage that we did not have control over the animals’ background. In addition, we did not receive any detailed information on age or interventions on the live animals prior to euthanasia. Such use of materials is justified because in the present study, we fully focus on paired, i.e. within-animal comparisons, with appropriate power to assess reproducibility and feasibility to accurately study differences between quasi-static and dynamic stiffness.

With the present findings we focus on the dynamic (pulsatile) loading aspects in the circumferential direction. Large arteries experience little to no axial deformation during the cardiac cycle. The vessels might move in longitudinal direction, but vessels mostly have grown and remodelled to have no change in axial force with increasing pressures at in vivo stretch^46–50^. From a biomechanical point of view, it might be interesting to include axial dynamic measurements in an experimental set-up. However, with the present set-up this is not possible.

In our current experiments, by design, there was no intraluminal flow through the vessels (Figs. 1, 2A). We do acknowledge that in vivo, wall shear stress is an important determinant of endothelial-derived factors that regulate vascular tone which influences long-term remodelling^51^. However, our set-up is not built to study long-term structural changes but to characterise biomechanical arterial wall properties under pulsatile pressure loading. Developing a system that, besides a realistic pressure loading, also simultaneously shows a realistic flow loading profile (i.e. at murine pulse rate, with solution of appropriate viscosity) is not trivial^20^ and, to our knowledge, has not been performed for murine-size vessels. Giezeman et al. designed an elegant set-up enabling a steady-state flow independent of sinusoidal variations in transmural pressure. However, as detailed and discussed (see “Choice of pressure measurement location to represent intravascular pressure” in “Methods” section, and “Dynamic vs. quasi-static experiments” in “Discussion” section) pipette flow through a pipette will cause errors in the measurement of the transmural pressure waveform, which are of critical importance when assessing visco-elastic vessel properties.

### Outlook and relevance

Blood pressure and age are established as two major determinants of arterial stiffness^52^. There remains an ongoing discussion on the effect of heart rate on vascular stiffness^42,45,53,54^. In the present study, we were able to show the feasibility of our set-up for quantifying blood pressure- and loading rate-dependencies of arterial stiffness. The role of heart rate on arterial stiffness measurements can be further elaborated within this set-up.

In future, data generated using our set-up can be used to characterise constitutive models of the artery wall^12,55–57^. Such models can be used to interpret the obtained measurements, yielding insight into the mechanical behaviour and contributions of the individual wall components. Such analyses may contribute to better understanding of factors that contribute to arterial stiffness, which may eventually result in novel treatment opportunities.

The set-up we developed fits directly under a two-photon laser scanning microscope. This would allow imaging and quantification of structural wall components under the same loading conditions as our mechanical testing. Results from such measurements, hence, can potentially be used to directly inform mechanical (constitutive) models at the corresponding loading states, enabling precise studies of the structure–function relationships across the tissue and vessel scales.

### Conclusions

We demonstrated the feasibility of our integrated set-up for ex vivo biomechanical characterisation of passive biaxially-loaded murine carotid arteries under pulsatile conditions, with an acceptable reproducibility for practical application. In the present study, we have extended to murine carotid arteries the observation known from studies in other animals that arteries behave stiffer under pulsatile (in vivo) conditions than under quasi-static conditions. This set-up enables further detailed research on how vessel wall components affect arterial biomechanics. This knowledge is important in unravelling (abnormal) arterial stiffening, and associated cardiovascular consequences.

## Methods

### Experimental set-up

Our experimental set-up (Fig. 1) is designed to assess biomechanical characteristics of murine carotid arteries and other blood vessels of similar dimensions (i.e. loaded length ~ 8 mm, loaded diameter ~ 0.7 mm), under quasi-static as well as pulsatile conditions. The set-up is a closed-loop system, which enables it to run for a prolonged period of time to cyclically distend the mounted artery while re-using fluid for distension.

#### Fluid circuit

The bulk of the system’s fluid is contained in the vessel bath (~ 65 ml) and the fluid reservoir (~ 15 ml). Both are temperature controlled to 37 °C. Reservoir fluid is pressurised using compressed air; air pressure is regulated using a computer-controlled regulator (Bronkhorst IQ + FLOW, Bronkhorst High-tech BV, Ruurlo, The Netherlands; interfaced through universal serial bus (USB)). Reservoir pressure is measured using a pressure sensor (*P*_0_, Fig. 1) and determines the maximum steady-state transmural pressure of the mounted vessel segment; fluid in the vessel bath is exposed to ambient air and hence at atmospheric pressure. An optical level sensor detects when reservoir fluid lever drops below ~ 60%, starting the return pump to draw fluid from the vessel bath back into the reservoir.

From the reservoir, a short pipe leads to a compliant tube, indenter and a pulse generator (Fig. 1). With each pulse a small volume of fluid flows from the reservoir into a hydraulic resistance-compliance (RC) circuit. The main fluid resistance of this circuit (Fig. 1) is governed by a small multi-turn valve; through this valve fluid flows into the vessel bath. System compliance is distributed and arises from the use of compliant silicone tubing throughout the system (Fig. 1). The resulting pressure waveform is sharply rising and reasonably similar to an in vivo pressure waveform (Fig. 3). The RC circuit is connected to the proximal pipette (p, Fig. 1) via a flow-through pressure sensor (*P*_1_, Fig. 1). The distal pipette (d, Fig. 1) is connected to another flow-through pressure sensor (*P*_*2*_), of which the outflow end is closed during experiments. By de-airing and closing the outflow end of *P*_*2*_, there is no flow across the distal pipette, rendering the distal pipette to act as an extension of *P*_2_, causing *P*_2_ to capture intraluminal pressure with negligible phase and amplitude errors over a large bandwidth (also see “Lumped-parameter modelling of pressure sensor configurations” section below).

#### Vessel bath and ultrasound

The two custom-drawn glass pipettes (outer diameter ~ 400 µm) between which the vessel segment is mounted, are submerged in the vessel bath. The proximal pipette (p, Fig. 1) is attached to a slide, allowing motorised axial stretch setting (by stepper motor). The distal pipette (d, Fig. 1) is attached to a load cell, facilitating axial force measurement.

Mounted vessels are imaged longitudinally from the top using a high-frequency ultrasound transducer (MS700, FujiFilm VisualSonics Inc., Toronto, ON, Canada), connected to a VEVO 2100 system. The transducer was fixed in a holder throughout the protocol, ensuring imaging plane consistency between measurements. Acquisition was performed in free running (no triggering) B-mode, which, using our settings (Table S4), resulted in a frame rate of 564 Hz. For each pulsatile recording, 1000 consecutive frames are acquired.

#### Control of indenter and motorised slide

An Arduino UNO microcontroller (Arduino, Somerville, MA, USA; interfaced through USB) is used to control the pulse generator and stepper motor (axial stretch). In addition, synchronously with each pressure pulse, the microcontroller generates an electrical synchronisation pulse (see “Signal acquisition and control” section).

#### Signal acquisition and control

The following analogue signals are acquired by a USB-6001 A/D-D/A converter (National Instruments Corporation, Austin, TX, USA; interfaced through USB) at a sampling rate of 2500 Hz: pressures *P*_0_, *P*_1_, and *P*_2_; temperatures of reservoir and vessel bath; axial force; and the synchronisation signal. The level sensor is connected to a digital input of the A/D-D/A converter; the heating circuits of the reservoir and vessel bath, and the return pump are driven from three digital outputs.

Signal acquisition and control are performed through a custom LabVIEW interface (LabVIEW 2013, National Instruments Corporation, Austin, TX, USA). Importantly, the synchronisation signal is also fed to the ECG input of the VEVO 2100 ultrasound system, facilitating synchronisation of pressure signals and ultrasound acquisitions.

### Lumped-parameter modelling of pressure sensor configurations

We performed lumped-parameter modelling to substantiate the relevance of our pipette and pressure recording configuration, in conjunction with zero-phase error processing of pressure and diameter signals. The simulation code is included in Supplemental Digital Content 1. Parameterisation was as follows: Proximal pipette flow resistance was determined from timed volume collection at a known pressure gradient and was about 1 × 10^11^ Pa/(m^3^/s). From preliminary experiments we estimated the compliance of a vessel under test to be about 5 × 10^–14^ m^3^/Pa at ‘normal’ physiological pressure conditions, but considerably greater at low pressure conditions: about threefold. The resultant *RC* time under physiological pressure conditions hence was 5 ms (simulation code in Supplemental Digital Content 1: in the model this is parameterised by an arbitrary *R*_pipette_prox_ = *R*_pipette_dist_ of 1 and a *C*_vessel_ of 0.005). Using *f*_c_ = 1/(2π*RC*) yields a corner frequency of 32 Hz.

### Experimental procedures

**Figure 8:**
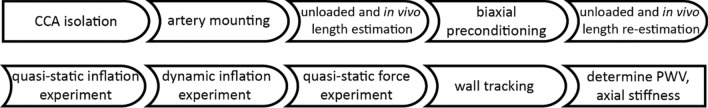
Timeline of experimental protocol. *CCA* common carotid artery, *PWV* pulse wave velocity.

An overview of the experimental procedure can be found in Fig. 8.

#### Animals

We obtained left and right CCAs from 11 male surplus mice, received from the animal facility of Maastricht University. Animals were euthanised with an overdose of carbon dioxide, after which both CCAs were isolated. One CCA was directly tested, while the other CCA was stored in Hanks’ Balanced Salt Solution (HBSS; Thermo Fisher Scientific, Paisley, UK) at 3 °C for testing the next day (maximum storage time was 24 h). We started with the left and right CCA in alternation. All experiments were performed in duplicate, requiring between 3 and 4 h per vessel. All methods were carried out in accordance with relevant guidelines and regulations. The use of surplus animals, after euthanasia, has been approved by the Maastricht University Animal Ethical Committee.

#### Preparations

To prime the system, the vessel bath was filled with HBSS, with the return pump used to pump this fluid into the reservoir. To achieve maximal vasodilatation, 10 μM of SNP (Sigma-Aldrich, St. Louis, MO, USA) was added to the vessel bath. After filling the entire system, air bubbles were removed and the dead-end tubing of *P*_2_ was closed off to ensure null compliance distal to the distal pipette (Fig. 1).

#### Preconditioning

After priming the circuit and mounting the vessel, the vessel was set to its unloaded length. This was done by increasing the length of the mounted vessel in 100-µm steps until the segment appeared straight (i.e. no visually obvious buckling, but not under tension), at negligible transmural pressure. Axial force was then calibrated to read zero. Subsequently, an estimate of the axial in vivo stretch was determined during preconditioning (${\uplambda }_{{z,iv,{\text{prec}}}}$) as the stretch at which axial force remains constant during pressurisation from 0 to 140 mmHg^36,37,58^*.* To eliminate excessive hysteresis and restore wall component rearrangements after excision, the artery was pre-conditioned circumferentially (4 inflation-deflation cycles from 0 to 200 mmHg; cycle time ~ 10 s) and axially (4 stretch-relaxation cycles between force zero and $F_{max,\text{prec}}$; step size 50 μm*; P*_2_ = 100 mmHg) before the start of the experiment^18,27,37,59,60^. $F_{{{\text{max}},{\text{prec}}}}$ was determined as the measured force at ${\uplambda }_{z} = 1.05{\uplambda }_{{z,iv,{\text{prec}}}}$ and $P_{2} = 200\;{\text{mmHg}}$. After this two-step pre-conditioning protocol, the resting length and in vivo axial stretch (${\uplambda }_{z,iv}$) were re-estimated using the method described above. This (re-estimated) ${\uplambda }_{z,iv}$ was used as a starting point for further experiments (Fig. 8).

#### Quasi-static inflation experiments

Initially, the artery was tested quasi-statically at ${\uplambda }_{z} = {\uplambda }_{z,iv}$ by measuring inner diameter at small pressure increments from 0 to 200 mmHg and back to 0 mmHg, in steps of 5 mmHg. Time between steps was ~ 5 s. We repeated the experiment with ${\uplambda }_{z}$ decreased by 5% (${\uplambda }_{z} = 0.95{\uplambda }_{z,iv}$) and increased by 5% (${\uplambda }_{z} = 1.05{\uplambda }_{z,iv}$). During the experiment at $1.05{\uplambda }_{z,iv}$, the axial force at $P_{2} = 200\;{\text{mmHg}}$ was recorded as $F_{{{\text{max}}}}$ (similar to the preconditioning protocol; see above).

#### Dynamic inflation experiments

To start the second experiment, ${\uplambda }_{z}$ was set back to ${\uplambda }_{z,iv}$. Subsequently, the pulse generator was activated at 5 Hz (300 bpm). Reservoir pressure and fluid resistance were tuned such that the maximum and minimum pressure of each pulse cycle as recorded by *P*_2_ were approximately 120 and 80 mmHg, respectively. Vessel distension was simultaneously recorded through ultrasound. After this measurement, reservoir pressure was tuned to result in maximum pressures of 80 mmHg and 160 mmHg respectively, to record pulsatile artery distension at typical hypotensive and hypertensive peak pressures.

#### Quasi-static stretch experiments

Finally, axial stiffness was assessed quasi-statically, by measuring axial force at small ${\uplambda }_{z}$ steps (${{\Delta \uplambda }}_{z}$). Axial stretch was increased from resting stretch (i.e. ${\uplambda }_{z}$ for which $F = 0$) up to $F_{{{\text{max}}}}$ in steps of ${{\Delta \uplambda }}_{z} = 0.015$ and then decreased back to resting stretch with the same step size. This experiment was performed at static transmural pressures of 10, 60, 100, 140, and 200 mmHg. Diameter assessment was not possible during these measurements as the ultrasound probe did not physically fit between the micropipettes at the resting length. Hence, we did not obtain diameter information in this part of the protocol.

### Data processing and calculations

B-mode ultrasound images were processed using B-mode edge tracking as described by Steinbuch et al.^61^. Dynamic pressure and diameter signals were resampled off-line at 1000 Hz. Data were filtered using a 51-point Savitzky-Golay filter of order 8 (*N* = 8, *M* = 25), having a − 3 dB cut-off frequency of ~ 60 Hz^62^. At a pulse frequency of 5 Hz, this leads to inclusion of the first 12 harmonics of the signals, yielding an accurate representation of the actual signals^63,64^.

We chose to quantify structural vascular stiffness by means of the pulse wave velocity (PWV) because this allows comparison with in vivo vascular stiffness measures. PWV was derived from the Bramwell–Hill relationship:

1 $${\text{PWV}} = \sqrt {\frac{1}{2{\uprho}} \times \frac{P_{\text{max}} - P_{\text{min}} }{d_{\text{max}} - d_{\text{min}}} \times d_{\text{min}}} ,$$

with ρ the blood mass density, *P*_min_ and *P*_max_ the minimum and maximum pressures, and *d*_min_ and *d*_max_ the minimum and maximum inner diameters (Fig. 7). Vessel inner diameter was determined with an operator-independent MATLAB script (MATLAB R2019a, MathWorks, Natick, MA, USA), to trace the exact location of the complete vessel wall for every timeframe^65^.

Because distensibility and compliance are measures often reported in ex vivo studies, we calculated those for the quasi-static—as well as the dynamic inflation experiments. However, repeatability statistics were only performed with our main outcome variable, PWV.

The distensibility coefficient was calculated as:

2 $${\text{DC}} = 2 \times \frac{{d_{{{\text{max}}}} - d_{{{\text{min}}}} }}{{d_{{{\text{min}}}} \times \left( {P_{{{\text{max}}}} - P_{{{\text{min}}}} } \right)}}.$$

Compliance coefficient was calculated from:

3 $${\text{CC}} = \frac{{d_{{{\text{max}}}} - d_{{{\text{min}}}} }}{{P_{{{\text{max}}}} - P_{{{\text{min}}}} }}.$$

To quantify arterial structural stiffness in the axial direction, an in vivo axial stiffness coefficient $c_{{{\text{ax}}}}$ (in grams) was calculated as the local derivative of the force-stretch curve for every static pressure:

4 $$c_{\text{ax}} = {\frac{{\text{d}}F}{{\text{d}}}\uplambda_{z} } \Bigg|_{{\uplambda }_{z} = {\uplambda }_{z,iv,{\text{calc}}} }.$$

The in vivo axial stretch value used in this equation (${\uplambda }_{{z,iv,{\text{calc}}}}$) was determined as the mean of crossing points of the quasi-static force-stretch curves at 60, 100, 140, and 200 mmHg (Fig. 6^11^). The force-stretch curve at 10 mmHg morphologically differs substantially from the other curves, and was therefore not included.

Data was processed with a custom MATLAB code.

### Statistical analysis

Set-up repeatability was assessed by quantifying within-subject and between-subject SDs and CVs. Within-subject SD was estimated by (1) calculating the sample SD for each set of (two) replicate measurements, (2) averaging the square of these SDs, and (3) taking the square root of the result^66,67^. Subsequently, between-subject SD was estimated in two steps. First, the SD of the subject means was calculated ($s_{{\overline{x}}}$). Note that the value of $s_{{\overline{x}}}$ is a measure of an inherent “component” of variation between subjects, and in addition, a “component” owing to the measurement error within each subject^67^. Hence, second, between-subject SD ($s_{{\text{b}}}$) was calculated as

5 $$s_{{\text{b}}} = \sqrt {s_{{\overline{x}}}^{2} - \frac{{s_{{\text{w}}}^{2} }}{2}} .$$

Sample size calculations were performed using the MATLAB Statistics and Machine Learning Toolbox at power 1−β = 0.80 and α = 0.05. Data between duplicate measurements of the same vessel were compared with a Wilcoxon signed-rank test for paired measures. To explore the potential influence of vessel freshness and left/right CCA on the outcomes, data from fresh/non-fresh and left/right CCA vessels were compared using a Mann–Whitney U test. All results are expressed as median with corresponding 25th and 75th percentiles and *n* denoting the number of vessels per group. *p* < 0.05 was considered statistically significant. Statistical analyses were performed using IBM SPSS Statistics for Windows (version 24.0; IBM Corp, Armonk, NY).

## Supplementary Information

Supplementary Information.
