# Supplementary material for: An integrated set-up for ex vivo characterisation of biaxial murine artery biomechanics under pulsatile conditions
Source: Sci Rep. 2021 Jan 29;11:2671. doi: 10.1038/s41598-021-81151-5 (PMC7846753; doi:10.1038/s41598-021-81151-5)
Supplement: Supplementary file 1 — Supplementary Information. [file 41598_2021_81151_MOESM1_ESM.docx]

An integrated set-up for *ex vivo* characterisation of biaxial murine artery biomechanics under pulsatile conditions

Myrthe M. van der Bruggen, Koen D. Reesink, Paul J.M. Spronck, Nicole Bitsch, Jeroen Hameleers, Remco T.A. Megens, Casper G. Schalkwijk, Tammo Delhaas, and Bart Spronck

# Supplemental Digital Content 1

# Corresponding author

Bart Spronck, PhD
Department of Biomedical Engineering
CARIM School for Cardiovascular Diseases
Maastricht University
Universiteitssingel 50, Room 3.359
6229ER, Maastricht
The Netherlands
+31 43 388 1659
b.spronck@maastrichtuniversity.nl

# Supplemental Tables

**Table S1.** Quasi-static single-point pulse wave velocity (PWV, m/s) at pressure range 120/80 mmHg determined at *in vivo* axial stretch ($\lambda_{z}=\lambda_{z,iv}$) and at 5% above and below *in vivo* axial stretch.

| **Quasi-static inflation pulse wave velocity (PWV)** | | | | | | |
| --- | --- | --- | --- | --- | --- | --- |
| $\lambda_{z}$ | Measurement 1 | Measurement 2 | Fresh | Non-fresh | Left | Right |
| $0.95\lambda_{z,iv}$ | 2.1[2.0-2.2] | 2.1[2.0-2.2] | 2.1[2.0-2.3] | 2.1[2.0-2.2] | 2.1[2.0-2.3] | 2.1[2.0-2.2] |
| $1.00\lambda_{z,iv}$ | 4.9[3.8-5.3] | 5.0[4.0-5.8] | 4.5[4.2-5.4] | 5.3[4.1-5.7] | 4.4[4.1-4.9] | 5.4[4.7-5.8]* |
| $1.05\lambda_{z,iv}$ | 5.1[4.1-5.6] | 5.4[4.3-6.0] | 4.6[4.2-5.3] | 5.6[4.7-6.2] | 4.7[4.3-5.6] | 5.5[4.4-5.9] |

Values denote medians [25^th^-75^th^ percentile]. *n=*20 for measurements 1 and 2*, n*=10 for fresh/non-fresh and left-right comparisons, **p*<0.05 for statistically significant differences. PWV based on the Bramwell-Hill equation. Differences between measurement 1 and 2 were not statistically significant (Wilcoxon signed rank test; *p*=0.65, *p*=0.40, *p*=0.27 respectively). Fresh and non-fresh vessels did not yield different PWVs for the different stretches (Mann-Whitney test; *p*=0.80, *p*=0.65, *p*=0.052). PWV did not differ between left and right carotid arteries for $\lambda_{z}=0.95\lambda_{z,iv}$ and $1.05\lambda_{z,iv}$ (Mann-Whitney test; *p*=0.58, *p*=0.39). It did however for $\lambda_{z}=\lambda_{z,iv}$ (*p=*0.04).

**Table S2.** Single point pulse wave velocity (PWV, m/s) for quasi-static and dynamic pressure-diameter relations at maximum pressure of 80, 120, and 160 mmHg.

| **Dynamic inflation pulse wave velocity (PWV)** | | | | | | | |
| --- | --- | --- | --- | --- | --- | --- | --- |
| Pressure  [mmHg] | Measurement 1 | Measurement 2 | Fresh | | Non-fresh | Left | Right |
| 80 | 2.4[2.2-2.5] | 2.3[2.2-2.4] | 2.4[2.3-2.6] | | 2.3[2.2-2.4] | 2.4[2.3-2.6] | 2.3[2.2-2.4] |
| 120 | 5.7[5.1-6.5] | 6.3[4.8-7.5] | 5.7[5.2-6.4] | | 5.9[5.5-7.0] | 5.9[5.2-7.1] | 5.9[5.6-6.3] |
| 160 | 14.8[11.5-16.0] | 13.9[11.6-15.7] | 14.2[11.6-15.6] | | 14.5[11.4-15.7] | 14.0[11.4-15.5] | 14.7[11.5-15.7] |
|  | | | | | | | |
| **Quasi-static inflation pulse wave velocity (PWV)** | | | | | | | |
| Pressure  [mmHg] | Measurement 1 | Measurement 2 | | Fresh | Non-fresh | Left | Right |
| 80 | 2.1[1.9-2.2] | 2.0[1.9-2.2] | | 2.1[1.9-2.3] | 2.1[2.0-2.2] | 2.1[1.9-2.2] | 2.1[2.0-2.2] |
| 120 | 4.9[3.8-5.3] | 5.0[4.0-5.8] | | 4.5[4.2-5.4] | 5.3[4.1-5.7] | 4.4[4.1-4.9] | 5.4[4.7-5.8]* |
| 160 | 11.4[9.8-12.6] | 11.3[9.5-12.4] | | 10.7[9.2-12.9] | 11.3[10.0-12.2] | 10.0[9.7-11.8] | 11.6[10.2-12.6] |

Values denote medians [25^th^-75^th^ percentile]. *n=*20 for measurements 1 and 2*, n*=10 for fresh/non-fresh and left-right comparisons, **p*<0.05 for statistically significant differences. PWV was calculated using the Bramwell-Hill equation. Maximum pressures were predefined; minimum pressures were derived from the dynamic experiments. Quasi-static PWV was determined at $\lambda_{z}=\lambda_{z,iv}$, for pressure ranges as obtained from the dynamic experiments. Duplicate measures did not statistically differ for the dynamic (*p*=0.47, *p*=0.36, *p*=0.68) and quasi-static (Wilcoxon signed rank test; *p*=0.40, *p*=0.40, *p*=0.56) experiments. Vessel freshness did not influence the results (Mann-Whitney test; Fresh vs. non-fresh; dynamic; *p*=0.28, P=0.86, *p*=0.47. Quasi-static; *p*=0.65, *p*=1.00, *p*=0.65). PWV differed significantly between left and right carotid artery in the group with a maximum pressure of 120 mmHg (Mann-Whitney test; dynamic; *p*=0.13, *p*=0.86, *p*=0.70. Static: *p*=0.65, *p=*0.04, *p*=0.39).

**Table S3.** Axial stiffness coefficient (g) for quasi-static pressure-diameter relations.

| **Quasi-static axial stiffness coefficient (c_ax_)** | | | | | | |
| --- | --- | --- | --- | --- | --- | --- |
| Pressure [mmHg] | Measurement 1 | Measurement 2 | Fresh | Non-fresh | Left | Right |
| 10 | 1.3[1.0-1.5] | 1.3[1.1-1.6] | 1.3[1.1-1.4] | 1.4[1.1-1.5] | 1.4[1.0-1.5] | 1.3[1.1-1.3] |
| 60 | 1.3[1.2-1.5] | 1.2[1.1-1.4] | 1.3[1.2-1.4] | 1.3[1.2-1.5] | 1.3[1.1-1.5] | 1.3[1.2-1.4] |
| 100 | 2.5[2.1-2.7] | 2.9[2.3-3.0]* | 2.7[2.2-3.1] | 2.5[2.1-2.8] | 2.6[2.1-2.9] | 2.6[2.2-2.9] |
| 140 | 5.2[4.3-6.3] | 5.1[4.4-6.0] | 5.5[4.4-5.9] | 5.0[4.2-6.2] | 5.0[4.2-6.5] | 5.5[4.4-5.9] |
| 200 | 10.7[8.7-11.9] | 10.4[8.6-11.6] | 11.0[(8.3-12.9] | 10.3[8.6-11.3] | 11.1[8.6-13.4] | 10.5[8.3-11.2] |

Values denote medians [25^th^-75^th^ percentile]. *n=*20 for measurements 1 and 2*, n*=10 for fresh/non-fresh and left-right comparisons, **p*<0.05 for statistically significant differences. Axial stiffness coefficient was calculated as the local derivative of the force-stretch curve around the *in vivo* stretch. Duplicate measurements were significantly higher between duplicate measurements at 100 mmHg, but not between measurements at other pressures (Wilcoxon signed rank test; *p*=0.21, *p*=0.33, *p*=0.03, *p=*0.30, *p*=0.55). There was no difference between fresh and non-fresh vessels for each pressure (Mann-Whitney test; *p*=0.66, *p*=1.00, *p*=0.18, *p*=0.66, *p*=0.66), nor between left and right common carotid arteries (Mann-Whitney test; *p*=0.39, *p*=0.97, *p*=1.00, *p*=0.97, *p*=0.48).

**Table S4**. Ultrasound device (VisualSonics VEVO 2100) settings during quasi-static and dynamic experiments.

|  | Dynamic experiment | Static experiment |
| --- | --- | --- |
| Transmission |  |  |
| Frequency | 50 MHz | 50 MHz |
| Power | 100 % | 100 % |
| Acquisition |  |  |
| Gain | 20 dB | 20 dB |
| Frame Rate | **>500* Hz** | **5 Hz** |
| Depth | 7.00 mm | 7.00 mm |
| Width | 4.73 mm | 4.73 mm |
| Line Density | **Standard** | **High** |
| Persistence | Off | Off |
| Sensitivity | Standard | Standard |
| ECG/Respiratory Gate | Off/Off | Off/Off |
| Extended Buffer | **Off** | **On** |
| Display |  |  |
| Dynamic Range | 55 dB | 55 dB |
| Display Map | G5 | G5 |
| Other |  |  |
| Number of focal zones | 1 | 1 |
| Focus depth | With artery | With artery |

*free running mode. Effective resulting frame rate for dynamic experiments was 564 Hz. Settings that differed between static and dynamic experiments are boldfaced.

# Supplemental Figures

**
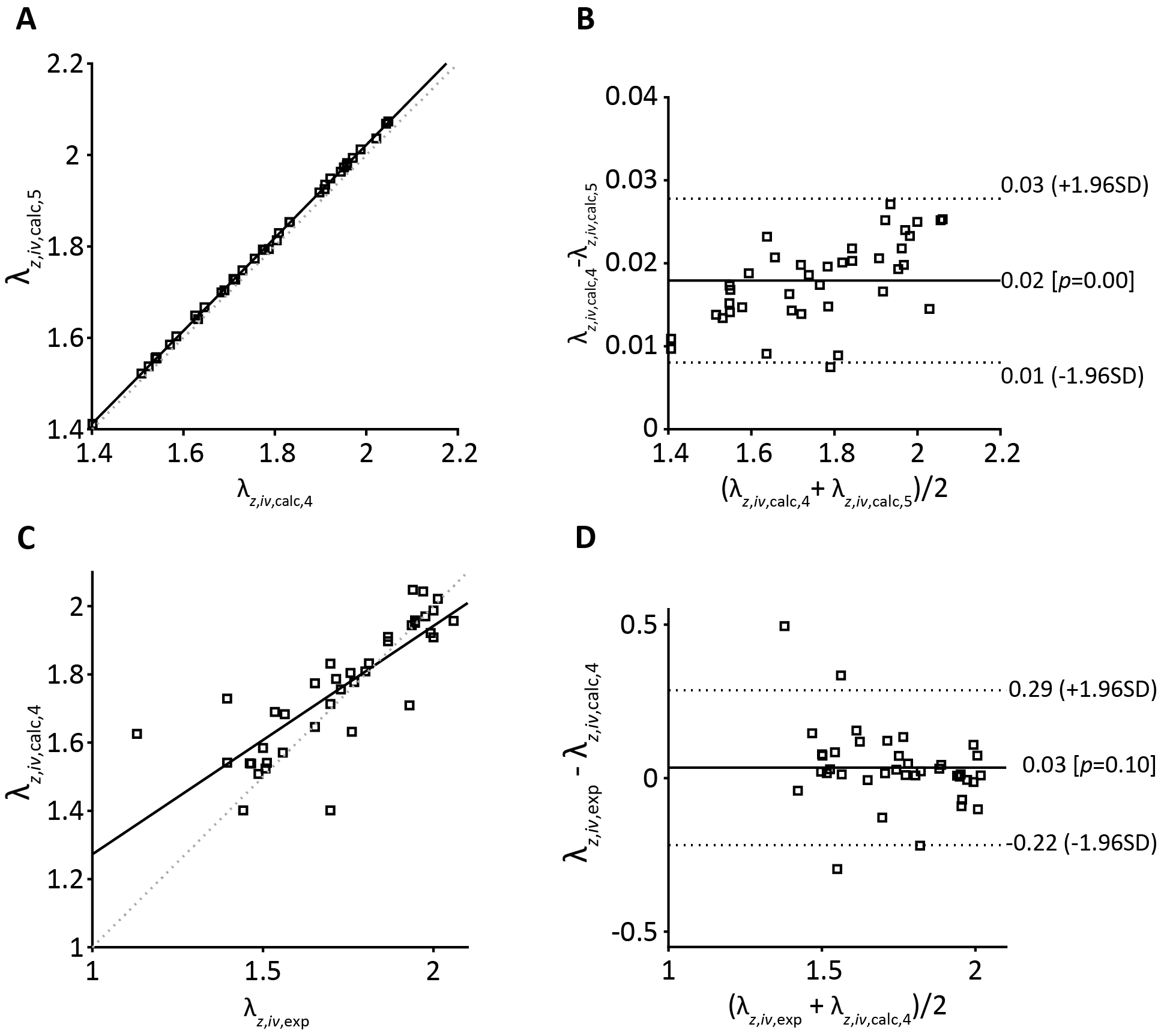
**

**Figure S1**. Method of in vivo stretch estimation. **A**, **B**: Comparison between *in vivo* axial stretch ($\lambda_{z,iv}$) as obtained using the crossing points of 4 force-stretch curves ($\lambda_{z,iv,calc,4}$; 60, 100, 140, and 200 mmHg), and 5 force-stretch curves ($\lambda_{z,iv,calc,5}$; 10, 60, 100, 140, and 200 mmHg). Note that $\lambda_{z,iv,calc,4}$ is termed $\lambda_{z,iv,calc}$ in the main text. In A and B, solid and dotted lines represent regression and identity lines, respectively. **C**, **D**: Difference in arterial *in vivo* axial stretch as estimated during the experiment ($\lambda_{z,iv,exp}$) and calculated post-hoc based on the force-stretch curves ($\lambda_{z,iv,calc,4}$, as in panels A and B). In C and D, solid and dotted lines represent the mean and 95% limit of agreement (defined as 1.96SD), respectively. SD, standard deviation.

# Simulation code for lumped-parameter modelling of pressure sensor configurations

* PSPICE Student / 91pspstu.exe

* Simulation scheme to characterize

* differences in phase shifts and amplitude transfers

* between P1 and P2 measurement locations to represent

* intravascular pressure Pvessel

* netlist notation is:

* modelElement        node1            node 2     value

Vpulse                P1               0          AC 1 0

* forms a pressure source with a unit sinusoidal amplitude

RproxPipette          P1              Pvessel   1

Cvessel              Pvessel          0          5m

* with these parameters the RC corner frequency is 32Hz (3db point)

* with the Cvessel being 3 times larger (15m) the RC corner

* frequency will be 10Hz

RdistPipette         Pvessel          P2         1

RclosedEnd             P2              0          1G

* RclosedEnd is virtually infinite, i.e. a million times Rpipette

.OP

.AC DEC 10 0.1 50

* the AC analysis sweeps the frequency from 0.1 to 50Hz and

* calculates the three node pressures

.PROBE

* enables plotting node pressures, named as V(P1) = pressure P1,

* etc. P(...) plots the phase

.END
